# Toxoplasma gondii Pneumonia in an Immunocompetent Host: A Case Report

**DOI:** 10.31729/jnma.5849

**Published:** 2021-01-31

**Authors:** Kumud Bhattarai, Anjal Bisht, Bhawesh Thapa, Ananta Bhakta Uprety

**Affiliations:** 1 Department of Medicine, Bir Hospital, Kathmandu, Nepal

**Keywords:** *immunocompromised host*, *pneumonia*, *protozoan*, *Toxoplasma gondii*

## Abstract

Toxoplasmosis is an infection caused by the intracellular protozoan Toxoplasma gondii. An acute infection caused by the protozoan is usually asymptomatic but some patients may go into a complicated course. Though it is a common pathogen of immunocompromised states as HIV AIDS, rarely it may present in an immunocompetent host as pneumonia. We report a 38 years old male who presented with fever with respiratory distress associated with inguinal lymphadenopathy and trans-aminitis. Toxoplasma pneumonia was diagnosed by clinico-radiological and immunological methods. The patient was treated with a specific antimicrobial agent. A high degree of suspicion for the diagnosis and initiation of specific therapy can be lifesaving to the patient that might be rewarding to the treating physicians.

## INTRODUCTION

Toxoplasma is an intracellular pathogen widely prevalent in most parts of the world. People get infected through the ingestion of food and water contaminated with feces of cats that contain the oocyst of Toxoplasma gondii.^1^ Acute infection is asymptomatic in most immunocompetent individuals. Rarely it may present as toxoplasma pneumonia.^2^ We hereby present a case that presented with acute onset febrile illness associated with respiratory distress and was later diagnosed with acute toxoplasmosis. This case report is intended to reinforce clinicians to keep wide differential diagnosis and use their clinical skills and knowledge to reach a diagnosis in the background of the disease being widely prevalent.

## CASE REPORT

Thirty-five years old Nepalese male, a recent returnee from Qatar, where he worked as an electrician for 2 years, presented with a high-grade fever of 1-week duration associated with dry cough with occasional production of minimal mucoid sputum without hemoptysis. He had progressive shortness of breath, MMRC grade II over 1 week period. He had no history of headache, photophobia, or altered sensorium. The patient denied any exposure to pets as cats or the use of raw meat. He had no prior comorbidities and there was no history of use of any immunosuppressants. The patient was admitted to the general medical ward in September 2018.

On general examination, the patient was febrile with a maximum recorded temperature of 104°F. He was tachypneic with a saturation of 88% in room air. He was slightly pale without icterus. There was no evidence of lymphadenopathy initially. Chest examination revealed the presence of crackles on the right lower axillary region. Cardiovascular and neurologic examinations were unremarkable. Per abdominal examination revealed non-tender hepatomegaly with a liver span of 18cm.

**Figure 1:**
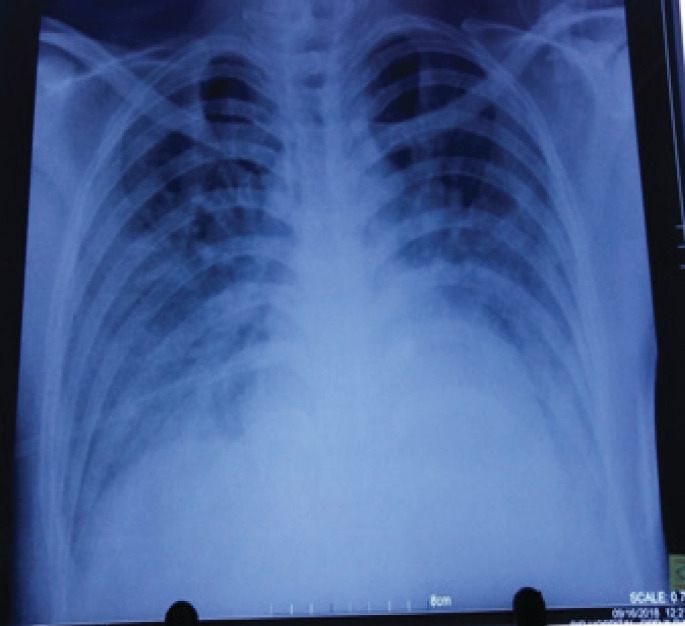
Chest radiograph showing reticular opacities prominent in bilateral mid and lower zones.

Investigations revealed: haemoglobin 10.7gm%, total counts 10400 with neutrophils 36% lymphocytes 64%, platelet 160000. The liver function test showed direct hyperbilirubinemia with transaminitis suggesting acute hepatitis. (total bilirubin: 2.4 mg/dl, direct bilirubin:0.8mg/dl, SGPT:397U/L, SGOT:235 U/L, ALP:186U/L). X-ray chest showed bilateral mid and lower zone infiltrates in a reticular pattern suggesting pneumonitis (Figure 1).

As the patient presented with fever and respiratory distress, with features suggestive of atypical pneumonia, a throat swab was sent to see any evidence of influenza. The RT PCR was negative for Influenza A and B infection. The patient was screened for HIV, Hepatitis A, B, C, and E, which were all negative. As the patient had daily spikes of high-grade continuous fever, tests for malaria, kala-azar, leptospirosis, scrub typhus, brucella, and blood culture were sent which were all negative.

After 2 days of stay in the general ward, the patient was shifted to ICU anticipating possible ventilator support given ongoing respiratory distress. His saturation was maintained only with high flow oxygen via face mask. The patient was empirically kept under piperacillin-tazobactam, levofloxacin, and supportive medications.

**Figure 2:**
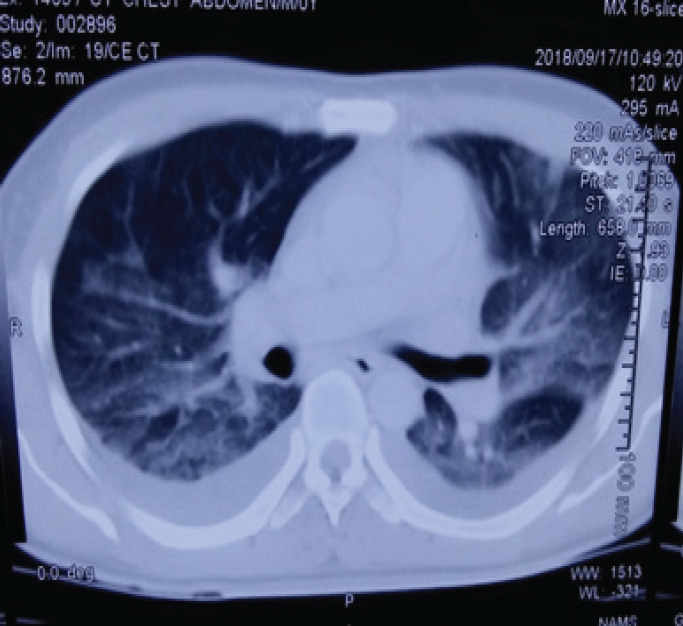
CT chest showing ground-glass opacities with bilateral minimal pleural effusion.

Saline nebulization induced sputum was negative for acid-fast bacilli and there was no growth of organisms in sputum culture. There were fever spikes even after 7 days of ICU stay. Clinical examination revealed the new onset of bilateral inguinal lymphadenopathy more prominent on the right side, the largest one measuring approximately 2*1 cm, nontender, nonmatted, freely mobile with normal overlying skin. No lymph nodes were palpable over the neck and axilla which was confirmed with ultrasound. CT chest was done that showed ground-glass opacities with bilateral minimal pleural effusion (Figure 2).

FNAC from the right inguinal lymph node showed a small number of lymphocytes and histiocytes. It was negative for any atypical cells or granuloma. Bone marrow aspiration and biopsy were performed which revealed normocellular marrow with the absence of granuloma or any parasites including LD bodies. ANA screening and Mantoux tests were negative.

Echocardiography was normal.

In view of acute febrile illness with pneumonitis, transaminitis with lymphadenopathy, serology for toxoplasmosis was sent. Toxoplasma IgM was high positive (>160IU/ml) with IgG 248 IU/ ml. The patient was started on cotrimoxazole (sulfomethoxazole+trimethoprim) on the 14^th^ day of admission. He became afebrile within 72 hours of this drug and dyspnea gradually improved. He was shifted to the general ward and later discharged with 4 weeks course of cotrimoxazole.

**Figure 3:**
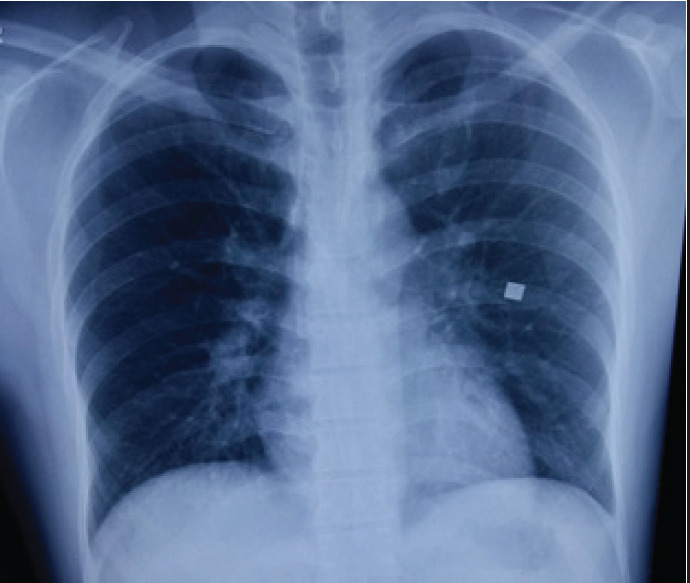
Follow up chest radiograph after 4 weeks showing radiological resolution.

Follow up visit after 4 weeks revealed complete clinical and radiological resolution (Figure 3).

## DISCUSSION

Toxoplasmosis is a protozoal infection caused by Toxoplasma gondii that is acquired in humans due to the ingestion of water and food contaminated with cat feces containing oocysts of Toxoplasma gondii. Other modes of acquisition is through ingestion of uncooked meat from infected animals that contain tissue cysts, through vertical transmission, and during organ transplantation.^1^ The clinical presentation can vary according to genotypes and geographical locations. Three genotypes (Type I, II, and III) of Toxoplasma have been described.^3^ The seropositivity for toxoplasma was found to be 11% in the age group 6-49 years in a study done in America.^4^ Similarly in a study done in some parts of Brazil, seroprevalence was up to 78%.^5^ Toxoplasma is thought to be infecting around 1/3^rd^ of the population globally making it one of the very successful parasites on the earth in terms of disease burden.^6^

Acute infection in most of the case is asymptomatic in immunocompetent individuals. Patients may present with asymptomatic generalized lymphadenopathy.^7^ However any or all of the lymph node groups can be involved.^1^ Severe form of illness can be in the form of pneumonitis,^2^ ARDS, hepatitis, or myocarditis.^8^ Sometimes it can also present as pyrexia of unknown origin. Toxoplasmosis in immunocompromised patients is mainly due to the reactivation of latent infection. These groups of patients present with life-threatening CNS infections including ring-enhancing lesions in the brain, encephalitis, chorioretinitis, pneumonitis, and multiorgan failure.

The diagnosis of toxoplasmosis is made by serologic and non-serologic methods. Serological tests include ELISA^9^, Indirect fluorescent antibody tests, and agglutination tests. IgM usually peaks in the first week of infection and gradually declines. However, IgG is usually detected after 2 weeks of infection and may persist for a lifetime. In immunocompromised hosts, reactivation of infection is indicated by the presence of positive IgG antibody which points towards some distant infection.^10^ Similarly non-serologic methods of diagnosis include the detection of organisms in different body fluids including blood, cerebrospinal fluid, aqueous humor, and bronchoalveolar lavage fluid. Polymerase chain reaction assays are sometimes used to detect the parasites’ DNA in different body fluids.^11^

Acute toxoplasmosis being the self-limiting illness, treatment is usually not indicated in immunocompetent individuals and non-pregnant women. However, in the case of severe forms of illness including pneumonitis, chorioretinitis, meningoencephalitis, treatment is indicated. The first line of therapy includes pyrimethamine 200mg loading dose then 1 mg/kg once daily plus sulfadiazine 1 gm four times a day with folinic acid 10-20 mg to prevent marrow suppression. The second line of treatment includes cotrimoxazole which is as effective as first-line therapy which is easily available. Treatment is generally given for 2-4 weeks depending upon the response.

Toxoplasma pneumonia is very rare in immunocompetent individuals. There should be a high degree of suspicion in the patient presenting with febrile illness with respiratory distress with associated lymphadenopathy and transaminitis. Clinical and radiological features are usually suggestive of atypical pneumonia. Prompt initiation of specific therapy can be lifesaving and rewarding to the treating physicians.
